# New Advances in Toxicological Forensic Analysis Using Mass Spectrometry Techniques

**DOI:** 10.1155/2018/4142527

**Published:** 2018-08-29

**Authors:** Noroska Gabriela Salazar Mogollón, Cristian Daniel Quiroz-Moreno, Paloma Santana Prata, Jose Rafael de Almeida, Amanda Sofía Cevallos, Roldán Torres-Guiérrez, Fabio Augusto

**Affiliations:** ^1^Ikiam-Universidad Regional Amazónica, Km 7 Via Muyuna, Tena, Napo, Ecuador; ^2^Institute of Chemistry, State University of Campinas, Cidade Universitária Zeferino Vaz, 13083-970 Campinas, SP, Brazil

## Abstract

This article reviews mass spectrometry methods in forensic toxicology for the identification and quantification of drugs of abuse in biological fluids, tissues, and synthetic samples, focusing on the methodologies most commonly used; it also discusses new methodologies in screening and target forensic analyses, as well as the evolution of instrumentation in mass spectrometry.

## 1. Introduction

The development of mass spectrometry methods has offered new possibilities for forensic toxicology analyses, where the identification and quantification of drugs of abuse are the most concerning issues in the forensic science [1]. The prevalence of drug addiction and abuse in the population worldwide is significantly high, resulting in one of the main causes of high criminal activities [2]. The excessive use of psychotropic substances, natural drugs, hallucinogens, and most recently “new psychoactive substances,” which are designed from skeletons of some natural drugs previously known, are the main focus of the development of new analytical methodologies, where mass spectrometry has had a key role [3, 4]. When a toxicological analysis needs to identify and quantify metabolites from unknown drugs, a screening can be performed by coupling different chromatography techniques, such as liquid and gas chromatography to mass spectrometry. In cases where an increment in the signal/noise ratio (S/N) is necessary, and the structure of the compound is known (target analysis) [5, 6] an additional selectivity can be provided using tandem mass spectrometry (MS/MS) in ion products or selected reaction monitoring (SRM). This latter one is the most widely used because of its increase in the specificity, selectivity, and detectability; however, the analyses become too time-consuming when a previous chromatographic separation and sample preparation are required [6, 7].

Moreover, ionization mass spectrometry techniques such as direct analysis in real time (DART), desorption electrospray ionization (DESI), low-temperature plasma (LTP), desorption atmospheric-pressure photoionization (DAPPI), paper spray (PS), touch spray mass spectrometry (TS-MS), more recently in toxicological analysis laser diode thermal desorption (LDTD), and atmospheric solids analysis probe (ASAP) have gained popularity as they can be used with less or even without sample preparation [8–10]. Nevertheless, depending on the matrix sample, compounds with identical patterns of fragmentation cannot be identified; this is the reason why more development in mass spectrometry needs to be conducted in order to provide relevant information that can help solve a crime [11]. In this sense, this review presents the main current applications of mass spectrometry for the control of drugs of abuse and the discovery of synthetic drugs in biological and synthetic matrices; besides, methodological limitations as well as innovative methodologies to enhance forensic toxicology analysis are discussed, examining the current literature in the past eight years.

## 2. Chromatography and Mass Spectrometry

### 2.1. Conventional MS Methods

The coupling of chromatography techniques with mass spectrometry has been widely used in drugs of abuse analysis, especially when the screening of the sample is needed, having separation techniques such as gas chromatography-mass spectrometry (GC-MS), liquid chromatography-mass spectrometry (LC-MS), liquid chromatography-mass spectrometry in tandem (LC-MS/MS) and, more recently, two-dimensional gas chromatography-MS (GC × GC-MS) as the most commonly used.

Normally, the analyses of nonobjective analytes, after the chromatographic separation, have the same steps to follow. First, a scan is performed by the mass spectrometer in order to identify or recognize some compounds of interest; then, it is necessary to perform a selected ion monitoring (SIM) [12, 13] in order to increase the sensitivity and selectivity of the analysis, in which only fragments of a specific group of molecules are monitored, resulting in an increased *S*/*N*. Consequently, this technique is the most widely used in quantitative analysis of compounds.

**Figure 1:**
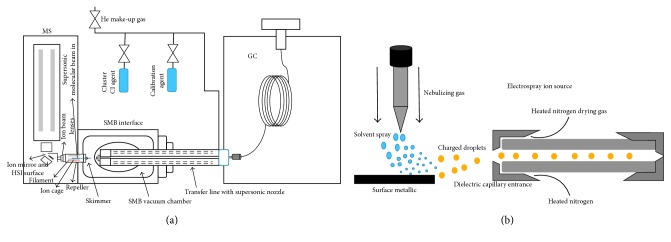
Main advances in mass spectrometry coupled to chromatographic technique in toxicological analysis are (a) gas chromatography interface-supersonic molecular beam with ionization vibration cold sample to mass spectrometry and (b) electrospray ionization and surface-activated chemical ionization to mass analyzed coupled with liquid chromatography.

The focus in this section is on the most recent and innovative analyses that have been performed using mass spectrometry coupled to chromatographic techniques, including all the new methodologies developed in toxicological analyses.  Table 1 provides a summary with their advantages and disadvantages, and Figure 1 shows these methodologies as well.

#### 2.1.1. GC-MS

The advances in techniques using MS coupled to gas chromatography have not been very significant due to the type of analyte that can be analyzed using this chromatographic technique (low molecular weight, volatiles). Even though high molecular weight compounds can be derivatized and analyzed by GC, the sample treatment is not appealing for the forensic toxicological analysis of drugs of abuse where the quickness of analysis is fundamental. For this reason, most of the advances using GC-MS focus on the resolution and separation capacity during the analysis. However, toxicological analysis methods in various matrices are well established and widely used in the analysis of drugs, in order to confirm forensic toxicology from samples of blood, urine, saliva, and hair, among others, during specific screening analysis, demonstrating high selectivity, detectability, and robustness.

In this sense, in order to improve the detection and identification of compounds using GC-MS, negative and positive modes of analysis in MS have been integrated, taking advantage of the stability of the fragments after a positive or negative ionization. For instance, Wu et al. used GC-MS with electron impact ionization and negative chemical ionization (GC/NCI-MS) and the traditional GC-MS with electron ionization mass spectrometry (GC/EI-MS) to analyze opiates, amphetamines, and ketamines in human hair. These analyses were capable of providing more sensitivity at low concentration of pictogram (pg) using only 25 mg from the sample and improving the detection of compounds during the analysis owing to the electronegative moieties. The strategy also avoided wrong results and misinterpretations obtaining lower limits of detection, in comparison with the use of only traditional GC/EI-MS in mode SIM; therefore, NCI can serve as a complementary technique in order to improve the sensitivity during the analysis [14].

The use of a miniaturized analytical method is the aim during the development of new analytical methods, and the analysis of drugs of abuse by mass spectrometry is not the exception. Most recently, GC-MS methodologies have used cold EI to analysis of heroin and cocaine [15, 16]. Here, the GC has an interface known as a supersonic molecular beam (SMB) where the ionization vibration cold sample is in an axial fly-through ion source configuration (Figure 1(a)), providing mass spectra with enhanced molecular ions that are compatible with reference libraries, and the range of compounds are amenable to GC-MS compounds. Additionally, this configuration allowed the increment of the flow rate in GC-MS without declines in the sensitivity in the analysis in the EI source, since the fly-through ion source sensitivity is fully independent on the column flow rate; therefore, column flow rate increase is automatically offset by a corresponding reduction in the helium make-up gas flow rate, the supersonic nozzle backing pressure, and the SMB flow rate are stabilized. The authors considered this aspect during the determination of heroin and cocaine in paper money and composite drug powders using column flow programming as a tool to further reduce the time of analysis. With this method, the time of analysis decreased, allowing the use of a column flow from 1 mL/min to 32 mL/min and the use of relatively small column dimension (5 m 0.25 mm) [55].

However, if the analysis aims to identify target compounds, and if specific fragments of a molecule are known, it is possible to increase the *S/N* with the use of mass spectrometry in tandem (MS/MS). GC-MS/MS is commonly used in SRM and *product ion scan* modes with collision-induced dissociation (CID). On the one hand, an ion precursor is generated into the collision cell during SRM mode, and then one ion product is monitored—this monitoring is also called transitions—this mode is widely used in quantitative analysis because of its selectivity. On the other hand, *product ion scan* consists of scanning product ions once the molecules are fragmented in the collision cell, generating, as a result, high reliability results due to the specificity of the monitored transitions. This method is generally used for transition optimization and the creation of libraries in MS/MS. Thus, these analyses can obtain an unequivocal identification of the eluted analyte. For example, this method identified methamphetamines in blood and urine with a simple and quick LLE and derivatization, as well as managed to differentiate between them [56].

Versace et al. used GC-MS to perform a screening of unknown compounds without an excessive sample preparation in urine samples and GC-MS/MS with the purpose of increasing the specificity using SRM transitions, identifying 54 drugs (i.e., 11-nor-9-carboxy-Δ^9^-tetrahydrocannabinol, cocaine, hydrocodone, and flurazepam) [57], while Emidio et al. developed a new methodology to determine cannabinoids in hair using 10 mg of sample and headspace solid-phase microextraction (HS-SPME) and GC ion trap/tandem mass spectrometry. Here, CID was used to adjust the breakage of the cannabinoid fragment (ion precursor) and improve the detectability of the technique, demonstrating an excellent linearity range between 0.1 and 8.0 ng/mg with a limit of quantification (LOQ) of 0.007–0.031 ng/mg and 0.012–0.062 ng/mg, which are smaller than the cutoff value established by the Society for Toxicological and Forensic Chemistry (GTFCh) [58].

On the other hand, ethyl glucuronides (a biomarker of ethanol), commonly used in the detection of chronic and excessive alcohol consumption, were identified using MS/MS operating in NCI-MS and SRM mode, obtaining differences between teetotalers and moderate drinkers, according to the current cutoff (i.e., 7 pg/mg hair). In this case, the use of negative mode provided an enhanced sensitivity in low concentration samples which were combined with the specificity of the fragments in the SRM analysis.

Therefore, a better analytical selectivity and S/N were achieved along with long-term markers for the detection of chronic and excessive alcohol consumption [6].

In the same manner, GC-MS/MS has been used to differentiate among important isomers such as methoxyethylamphetamines and monomethoxydimethylamphetamines, as synthetic drugs without derivatization. Using CID and SRM, the specificity of the fragments obtained provided intensity differences in product ions among the isomers, enabling mass spectrometric differentiation of the isomers [59]. One the contrary, GC × GC as a previous treatment can be used in order to increase sensitivity, detection, separation, and resolution. In this sense, GC × GC-MS was used in the determination of cannabinoid-like drugs in 1 mL of postmortem blood, which present a challenge due to the matrix interferences in endogenous lipophilic compounds, proteins, drug degradation/formation, and production of artefacts. With this technique, a limit of detection of 0.25 ng/mL for 11-hydroxy-Δ^9^-tetrahydrocannabinol was obtained [60].

The same methodology was applied in oral fluid samples, where it is common to have a small volume of samples, and the concentration of some drugs is usually lower, which can complicate the analysis [61]. The compounds were identified using GC × GC-MS with cold trapping and NCI-MS, obtaining a limit of detection of 0.5 ng/mL [61]. Additionally, GC × GC coupled with time-of-flight-MS (GC × GC-TOFMS) was used to analyze codeine, morphine, and amphetamines in sample extracts from hair suspected of containing various drug compounds. The analytical technique also identified metabolites such as cocaine, diazepam, and methaqualone, which are not included in the target analysis [62].

#### 2.1.2. LC

The use LC as a versatile separation technique (volatile and nonvolatile analytes) has improved the detection and quantification of analytes such as amphetamines, benzodiazepines, hallucinogens, cannabinoids, opiates, cocaine, designer drugs, pharmaceutical products, or illicit drugs in several matrices.

Ultrahigh-performance liquid chromatography has been used along with tandem mass spectrometry (UHPLC-MS/MS) operating in SRM mode in order to establish an individual ion transition ratio to each analyte. Thus, each analyte is infused into the electrospray capillary, and the declustering potential was adjusted to maximize the intensity of the protonated molecular species [M + H]^+^. The signals were optimized using a source block temperature of 500°C and an on-spray in the determination of Δ^9^-tetrahydrocannabinol, cannabidiol, and cannabinol in 50 mg of 179 hair samples. This method allowed the identification of one new synthetic cannabinoid, obtaining an LOQ of around 0.07 pg/mg and 18 pg/mg in the analysis [63].

High-resolution (HR) MS has been used successfully along with LC in the drug of abuse analysis. The resolving power and the high mass accuracy obtained with HRMS were advantageous in the analysis of complex matrices and data acquisition in a targeted and nontargeted manner in order to decrease the number of interferences caused by biological matrix in the drugs analysis. In this case, the authors used UHPLC-HR-TOFMS to analyze cannabinoids and cathinones in 1 mL of urine. During the analysis, a broad-band collision-induced dissociation (bbCID) was used with the purpose of providing a confirmation-level screening featuring both high sensitivity and wide scope. The precursor ions were fragmented in the collision cell without preselection, and the analysis allowed the identification of 75 compounds with cannabinoids spectra database with cutoff concentration values of 0.2–60 ng/mL and cathinones 0.7–15 ng/mL, respectively [64, 65].

The methods of ionization are fundamental to ensure the correct ionization of the sample and, therefore, the identification of the compounds, especially in complex matrices. Wang et al. performed an analysis of cocaine and their metabolites under three types of ionization such as electrospray ionization (ESI), atmospheric-pressure chemical ionization (APCI), and atmospheric-pressure photoionization (APPI) in order to evaluate the chemical suppression during the analysis of 17 illicit drugs in 100 *µ*L of oral fluids using UHPLC-MS/MS in mode SRM. The authors found that ESI presents the smallest ion suppression for all cocaine metabolites analyzed facing the APPI and APCI mode. However, the method developed obtained LOQs in ESI, APCI, and APPI in a range from 0.11 to 1.9 ng/mL, 0.02 to 2.2 ng/mL, and 0.02 to 2.1 ng/mL, respectively. The authors recommended further investigation to determine the causes of higher ion suppression in APCI and APPI on ESI in oral fluids, since ESI may suffer important matrix effects, as it is widely known. Nevertheless, they state that APCI and APPI probes evaporate inlet solutions and ionize analytes via gas-phase chemistry and, consequently, are less affected than ESI. For example, oral fluids may contain many salts and small molecules partitioned from plasma instead of macromolecules, which can lead to an increase in ion suppression in APCI and APPI. As a result, authors suggest the use of ESI in this type of analysis [66].

Liquid chromatography has also taken advantage the benefits of negative mode in mass spectrometry. In this case, LC coupled to (HR)-MS was used along with Orbitrap technology in the analysis of metabolites of drugs such as cocaine, ephedrine, and morphine in urine. The analyses were performed in full-scan mode with positive/negative switching, and subsequently making use of a selective screening through data dependent acquisition (DDA) mode, resulting in a fast analysis. Additionally, the risk of false-negative results caused by ion suppression or isomer overlapping could be reduced by including metabolites and artefacts, as well as recording in the positive and negative modes [67].

Recently, new methods of analyses in MS/MS have been coupled with LC techniques. Dynamic multiple reaction monitoring (dMRM) has been used in toxicological analysis, and it is recognized by the use of a timetable based on the retention time for each analyte. Such technique monitors the analytes only around the expected retention time, and decreases the number of concurrent SRM transitions, also known as multiple reaction monitoring (MRM), allowing both the cycle time and the dwell time to be optimized to the highest sensitivity, accuracy, and reproducibility [18]. For example, a quantitative LC-MS/MS method has been developed for the simultaneous determination of 17 antipsychotic drugs in human postmortem brain tissue; these drugs are of forensic interest because they have been associated with sudden death cases.

In this method, the analysis was performed operating on dMRM mode, using ESI+. Calibration curves prepared in the spiked brain tissue were linear in the range 20–8000 ng/g (*R*^2^ > 0.993) for all drugs, except olanzapine [19]. Besides, LC-MS/MS in dMRM mode was used by Shah et al. in order to identify around 200 drugs/metabolites, such as methamphetamine, amphetamines, ephedrine, and cocaine in hair samples [20]. This method proved an interesting alternative for fast analysis of drugs. All these analyses were performed in one chromatographic run (i.e., 8 min), showing a high sensitivity and accuracy [20].

With the purpose of identifying cannabinoids, such as Δ^9^-tetrahydrocannabinol, Conti et al. coupled LC along with two ionization systems, electrospray ionization, and surface-activated chemical ionization (ESI-SACI-MS) to several types of mass analyzer (ion trap, triple quadrupole, and Orbitrap) to improve the detection of 11-nor-9-carboxy-tetrahydrocannabinol in biological samples (urine and hair) operating in SRM mode. This coupling consists in a metallic surface that keeps a fixed voltage and that is inserted into a commercial ESI source (Figure 1(b)). This electrostatically charged surface is able to improve the ESI ionization efficiency, and it increases the ion focusing efficiency towards the mass spectrometric analyzer. The authors show that the sensitivity provided was better with SACI-ESI than with the classical ESI approach alone [17, 68].

Furthermore, an ultrafast and sensitive microflow liquid chromatography-MS/(MFLC-MS/MS) was used to quantify hallucinogens such as LSD and their metabolites in 500 *µ*L of plasma in order to miniaturize and accelerate the analysis of drugs of abuse using LC techniques; this coupling is known by its decrease in run numbers, a higher ionization yield, and reduced ion suppression/enhancement effects. Here, the MS ion trap operated in *product ion scan* and SRM mode in order to perform the quantification along with a dynamic fill-time trap; this method allowed sensitive detection and fast analysis, obtaining LOQs corresponding to 0.01 ng/mL for all analytes [69].

## 3. Current Analytical Approaches to Target Analyses

### 3.1. Mass Spectrometry

Mass spectrometry is the preferred technique when the aim is to perform a quick analysis directly on the sample. The main difference between chromatography-MS methods is the sample introduction. MS instrumentation is assembled by ion sources, mass analyzer, and detector [13]. However, the challenge in forensic analysis is the possibility of decreasing the time and cost of analysis per sample. A primary analytical focus in toxicology is determining the presence or absence of drug metabolites in biological samples. In this sense, the use of ambient ionization technique mass spectrometry has allowed the analysis of the entire sample without an excessive preparation of sample. These techniques make possible the concept of open-air surface analysis directly under ambient conditions, being particularly useful for surface analysis of solids, avoiding many, if not all, sample preparation steps typically required [8].

HRMS is widely employed in the coupling with ambient mass spectrometry currently because of its capability of measuring accurate masses and differentiating among compounds with identical nominal masses, providing a comprehensive full-scan MS and MS/MS for the search for any analyte without sample pretreatment. This provides accurate m/z values that can be used to generate chemical formulas with high mass accuracy (<5 ppm mass error) [70]. Therefore, HRMS can be theoretically applied in different configurations with interchangeable ionization sources and sophisticated data acquisition capabilities, making HRMS one of the preferred techniques for the analysis of new drugs [71].

**Figure 2:**
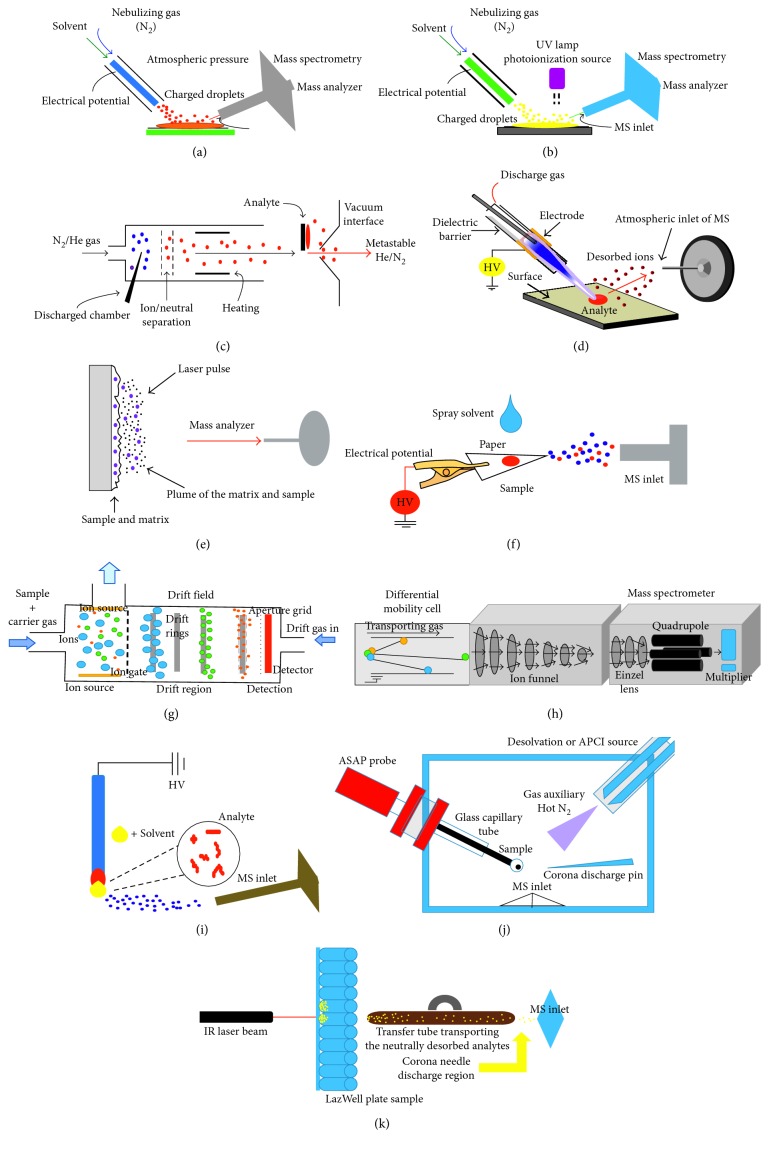
Main ambient ionization techniques used in toxicological forensic analysis. (a) DESI-MS, (b) DAPPI-MS, (c) DART-MS, (d) LTP-MS, (e) MALDI-MS, (f) PS-MS, (g) HPIMS, (h) IMS-MS, (i) TS, (j) ASAP-MS, and (k) LDTD-MS.

Ambient ionization mass spectrometry can be divided depending on the desorption technique, which will be discussed in the next section about the most used procedures in the analysis of drugs of abuse.  Table 1 and Figure 2 present the advantages and disadvantages of these techniques or procedures.

#### 3.1.1. Ambient Ionization Technique Mass Spectrometry: Desorption by Solid-Liquid Extraction

In these techniques, the desorption occurs by solid-liquid extraction followed by ESI-like ion-production mechanisms; the ionization can be performed by DESI (Figure 2(a)) or DAPPI (Figure 2(b)) [8].

DESI-MS is used in forensics due to its ability for in situ analysis. However, direct analyses that involve DESI and DAPPI are less common in toxicological analysis due to the interferences which can be caused by the suppression of ionization product of the matrix effects in the samples; therefore, adding an additional step in the preparation of the sample is often required. For instance, matrix-suppression effects were studied within direct analysis of benzodiazepines and opioids from 1 mg/mL of urine with DESI-MS and DAPPI-MS [21]. The authors found that the urine matrix affects the ionization mechanism of the opioids in DAPPI-MS and favors the proton transfer over charge exchange reaction. However, the sensitivity of the drugs in the solvent matrix was at the same level in DESI-MS and DAPPI-MS with limits of detection of 0.05–6 *µ*g/mL, along with a decrease in sensitivity for the urine matrix that was higher with DESI (typically 20–160-fold) than with DAPPI (typically 2–15-fold), indicating better matrix tolerance in DAPPI over DESI. This illustrates that urine contains high concentrations of salts in DAPPI, and the salts in the urine samples are not efficiently evaporated from the sampling surface which do not significantly interfere with the ionization [21].

DESI-MS also allowed the analysis of common drugs in urine samples such as pethidine, diphenhydramine, nortriptyline, and methadone using pretreatment sample by liquid-phase microextraction (LPME). These selective extraction capabilities of three-phase LPME provided a significant reduction in the matrix effects observed in direct aqueous LPME extracts [22]. However, some drugs such as Δ^9^-tetrahydrocannabinol and cannabidiol that have identical fragmentation spectra presented significant interference, resulting in the impossibility of an unequivocal identification of each other [22]. Additionally, DESI-MS/MS has been used coupled with solid-phase extraction (SPE) in order to analyze clenbuterol in urine specimens to detect doping; the authors mentioned that the suppression effects were minimized by SPE using DESI-MS/MS [23].

Moreover, DAPPI-MS has been used coupled with quadurople-ion trap MS and MS/MS mode to analyze directly herbal products such as *Catha edulis*, *Phycybe* mushoms, opium, designer drugs in tablets, confiscated drug samples of several forms as tablets, blotter paper, plant resin, and powder forms that contain meta-chlorophenylpiperazine, 3-fluoromethamphetamine, methylenedioxypyrovalerone, amphetamines, phenazepam, buprenorphine, and methylone. DAPPI-MS proved a specific analysis without sample preparation [24], showing that it is sufficient in most criminal cases where the main purpose is to do a qualitative screening [25].

#### 3.1.2. Thermal or Chemical Sputtering Neutral Desorption

These techniques involve metastable and reactive ions, in which the species react with the analyte directly or indirectly through proton- and charge-transfer reactions [8]. In the analysis of drugs of abuse, DART (Figure 2(c)) and LTP (Figure 2(d)) can be used; the former is the most preferred in toxicology forensic analysis and uses a negatively biased point-to-plane atmospheric-pressure glow discharge at lower currents, physically separated from the ionization region by one or several electrodes. The metastable species are formed within the discharge supporting gas that typically is He or N_2_, generating protonated water clusters. The main advantage of DART is the analysis of samples in solid, gas, and liquid states, handling polar and nonpolar analytes with masses below 1 kDa [26].

Compounds are identified by combining information about elemental compositions from exact masses and isotopic abundances with fragment-ion mass spectra obtained by collisional activation. In toxicological forensic analysis, DART has been widely used in the detection of small drugs, but its quantitation remains a big problem due to its minor reproducibility, which depends on the position of the sample inside the ion source, making the number of drugs that can be quantified very limited [27]. However, when it is necessary to obtain more details in the identification matrix based on the natural products, high temperatures of ionizing gas can be used during the analysis, benefitting the resolution of more complex spectra [28].

DART achieved the detection of *γ*-hydroxy butyrate without any sample preparation or other illicit synthetic cannabinoid products coupling mass spectrometry DART-MS with CID analysis. The use of fragments obtained by CID provided a sensitive and specific detection, increasing the limit of detection to identify individual components and showing the ions related to each synthetic cannabinoid, since the [M + H]^+^ precursor ions were still present in the mass spectra [29]. Thus, an unambiguous differentiation of each species could be accomplished. [M + H]^+^ precursor ions could also be used as a complement in the analysis of drugs through screening in order to identify new and unknown drugs.

DART-TOFMS detected alprazolam, which is one of the ingredients of the “Houston Cocktail,” containing hydrocodone/acetaminophen, and achieved an analysis with high mass accuracy [30]. Habala et al. identified six synthetic cannabinoids in methanolic extracts from solid herbal material using a DART source coupled with a hybrid ion trap—LTQ ORBITRAP—mass analyzer, discovering that the leaves have a greater concentration than the stems of the plant material [11].

In the identification of new psychoactive substances (NPS), DART-MS has had an important role. Gwak and Almirall [4] performed a screening of 35 NPS in urine using DART coupled with hybrid TOFMS and ion mobility spectrometry (IMS), identifying synthetic cathinones with a single phenethylamine as the most common compounds. The analytes detected had an error within ±5 ppm, but isomeric compounds could not be differentiated. Similarly, DART-TOFMS was used in order to detect synthetic cannabinoid in botanical matrices like *Coriandrum sativum*, *Ocimum basilicum*, and *Mentha spicata* [28]. In this research, intensive sample preparation was not required, just methanol dissolution, which is a method that allowed the identification of the synthetic cannabinoids such as AM-251 and JWH-015. Although botanical samples exhibit relatively complex mass spectral profiles, this did not prohibit the identification of the target compounds. Additionally, the DART-TOFMS analyses were conducted with different ionizing gas (helium) temperatures in order to determine the optimum desorption temperature, and it was observed that higher temperatures had the additional benefit of yielding more complex spectra that could permit a more detailed identification of the plant matrix based on the natural products [28].

Grange and Sovocool [31] developed a methodology for the extraction and clean-up of drugs in smoke deposited on household surfaces so as to determine the exposure of the patients to drugs of abuse using DART-TOFMS. A field sample carrier and an auto sampler were used to minimize the time per analysis. The sampling was performed just with cotton swab wipes with isopropanol, finding a quantification of each drug of around 0.025 *µ*g/100 cm^2^. However, Δ^9^-tetrahydrocannabinol and nicotine had m/z 315 and m/z 163 interferences, respectively. The authors found that this interference could be a sugar unit from the cellulose of the cotton-swabs. In spite of this interference, the method is highly recommended for the analysis of residues in clandestine drug laboratories [31].

Moreover, phenethylamine, a synthetic drug known by its effects similar to LSD and its sublingual consumption via blotter paper, was analyzed directly in the sample by DART-TOFMS. This was studied in blotter paper street samples, and the results can be used in preliminary identifications, since this technique is extremely fast and advantageous for the quick screening of unknown street samples in crime laboratories [32]. Poklis et al. used DART coupled with HRMS in the analysis of legal purchases on the Internet under the name “Raving Dragon Novelty Bath Salts and Raving Dragon Voodoo Dust” and found out that they contain methylone and pentedrone, respectively, which can be identified as unsupervised drug market [33].

More recently, DART has been developed using SPME-fiber format for coupling nanogold surfaces with mass spectrometry in order to perform an effective drug capture in toxicological matrices like methamphetamine, diazepan, and alprozolam in human plasma. The authors used LC-MS/MS and DART-MS/MS, in this case, coupling antibodies to nanogold-coated wires. An antibody with cross reactivity to multiple drugs was used for simultaneous extraction of a mixture of drugs. The immunoaffinity nanogold is known by its possibility of eliminating chemical noise. The limits of detection achieved with DART-MS/MS were comparable to those observed with LC-MS/MS [34].

Different mass analyzers have been used to evaluate sensitivity and selectivity in the detection of Δ^9^-tetrahydrocannabinol (THC) from intact hair samples using DART [35]. The mass analyzers evaluated were an Orbitrap, a quadrupole-Orbitrap, a triple quadrupole, and a quadrupole time-of-flight (QTOF). The authors found that only the quadrupole-Orbitrap in high-resolution mode achievement distinguished THC in hair samples from endogenous isobaric interferences [35]. Those are important data since when the resolution in the mass analyzers is low, the risk of obtaining false/positive is high.

Different from DART, LTP was developed for direct sampling ionization in chemical analysis using mass spectrometry. The plasma here is generated by dielectric barrier discharge (DBD) and a discharge of gas at low flow rate (<500 mL/min), and a high-voltage to sustain the plasma in an ambient environment [8]. This technique has proved a powerful tool in direct analyses, exclusively with small organic molecules with low to moderate polarity. For this reason, it is not commonly used in the analysis of illicit high molecular weight drugs as it limits the analysis of unknown drugs. However, LTP proved effective in the analysis of stomach fluid content of a diseased dog suspected to have died from ingestion of insecticide. Direct sampling ionization was applied in MS analysis and protonated Terbufos, and Terbufos sulfoxide were observed [72]. These two compounds are common in Terbufos-based insecticides, which were suspected to be the cause of the death of the dog.

Furthermore, the analysis of drugs of abuse in urine and 25 mg of hair extract samples were systematically investigated, where several drugs such as amphetamine, benzoylecgonine, caffeine, cannabidiol, cocaine, codeine, diazepam, ephedrine hydrochloride, heroin, ketamine, methadone, methamphetamine, morphine, and Δ^9^-tetrahydrocannabinol were identified obtaining a limit of detection of around 10 ng/mL without any sample preparation [36].

#### 3.1.3. Laser Desorption/Ablation

In these techniques, the analytes are desorbed or ablated from a surface by an IR or UV laser with or without a matrix (Figure 2(e)). The sample is subsequently merged with an electrospray droplet cloud or a plasma stream, depending on the ionization source used [8]. When the source excites an exogenous matrix that cocrystallizes and has energy absorbent capabilities, it can coat the sample surface to be analyzed. Then, a laser adds excess energy to the matrix-sample complex, where the matrix absorbs laser energy to pass it to the sample and, finally, to produce ions from analytes; this technique is called matrix-assisted laser desorption electrospray ionization (MALDESI) [73]. In contrast to MALDESI, metal-assisted secondary ion mass spectrometry (MetA-SIMS) procedure adds small amounts of metals onto sample surface to enhance mass spectra analysis [42]. These two methods may provide an image coupled with ionization mass spectrometric imaging (MSI), which is a powerful technique to obtain spatial information (distribution) of compound mass spectra.

Porta et al. used MALDESI coupled to MSI in order to monitor the distribution of cocaine and its metabolites in 12 mL of extracts of intact single hair samples from chronic users. The acquisitions were performed applying rastering mode in the SRM mode on a MALDI triple quadrupole linear-fitted ion trap. The time of analysis of an intact single hair sample of 6 cm was of 6 min approximately. Cocaine and its metabolites were identified and quantified, and the results were obtained with a limit of detection of 5 ng/mL, becoming an excellent methodology to detect cocaine consumption [37]. In the same manner, matrix-assisted laser desorption/ionization-mass spectrometry imaging (MALDI-IMS) was used to rapidly screen longitudinally sectioned drug user hair samples for cocaine and its metabolites; using continuous raster imaging, the optimization of the spatial resolution and raster speed were performed on cocaine-contaminated intact hair samples. Besides, the MALDI-MS/MS images showed the distribution of the most abundant cocaine, using *product ion scan* as a mode of analyzing. With this method, it is possible to obtain mass spectra with the main fragment of the molecule target.

An SRM experiment was also performed using the “dynamic pixel” imaging method to screen for cocaine and a range of its metabolites, in order to differentiate between contaminated hairs and drug users. Therefore, these methods are important when the imaging information on drug distribution is necessary, for example, in human hair without extensive sample preparation, or when labelling techniques are required. However, it should be noted that it only provides qualitative data about administered drugs, through a pixelated representation [38]. MALDESI has also been coupled with HRMS during the identification of 74 drug samples which were detected using the ionic liquid matrix N,N diisopropylethylammonium *α*-cyanohydroxycinnamate. This method allowed the identification of new designer drugs, which come from the use of safrole as a precursor for the synthesis. Nevertheless, the result obtained presents weaker resolution and lower sensitivities, leading to lower peak intensities. The authors affirm that this limitation is a consequence of the matrix, since this can be related to the formation of adducts, but the matrix may be enhanced by adding specific cations and anions. Further investigations to improve ionization through matrix additives are still necessary in the field. Another limitation of this methodology is the impossibility to distinguish drug position isomers, such as methamphetamine and 4-methylamphetamine, as well as structural elucidation of unknown compounds. The authors recommend the combination between this methodology and bioinformatics software tools which provide untargeted compound searches, even if respective HRMS spectra are not included in a library just based on the precursor ion fingerprinting [39].

On the contrary, MALDI-MSI and MALDI-Fourier transform ion cyclotron resonance (FTICR-MS) have also been used for mapping and direct detection of methamphetamine in longitudinal sections of the single hair sample in positive mode, in which umbelliferone was used as a matrix. This matrix has the advantage of being hydrophobic and capable of assisting in the ionization of methamphetamine in hair. The authors observed that the detection and sensitivity provided by this matrix is higher than *α*-cyano-4-hydroxycinnamic acid (CHCA) or 2,5-dihydroxybenzoic acid (DHB). In addition, the distribution semi-quantitative of methamphetamine can be performed. This method enhances the detection and sensitivity of target drugs embedded in a hair matrix, achieving a detection level down to nanogram per milligram; for this reason, the authors compared the results with the obtained by LC-MS/MS, but in this case with less sample amount required [40].

More recently, Kernalléguen et al. [41] have made possible the semiquantification of cocaine and its metabolites (benzoylecgonine, cocaethylene, and ecgonine methyl ester) in hair, using microarrays for MS and MALDI-MS/MS. So far, it is well known that the inhomogeneous MALDI matrix crystallization and laser shot-to-shoot variability make the quantitation more difficult; therefore, the authors used a high-throughput MALDI method, along with an innovative high-density microarray for mass spectrometry (MAMS) technology. This technology consists of a sample preparation slide containing lanes of hydrophilic spots, and an automated slider which drags a sample droplet over several small spots, with the purpose of achieving homogeneous crystallization of the matrix-analyte mixture and, therefore, to a reproducible signal. In this manner, it was possible to establish a calendar of consumption in only 1 mg of hair with a great correlation, becoming an excellent methodology when urgent results are required [41].

However, metal MetA-SIMS was used to determine the differentiation between systemic exposure and external contamination that remains in the hair because of exposure to drugs after following the protocols of decontamination (hair wash) [43]. The authors reached a comparison of the results among MetA-SIMS, MALDESI-MS, and LC-MS/MS, showing that there is still cocaine detected after the washes of decontamination, using MetA-SIMS. MALDESI was in turn inefficient for forensic hair analysis since no cocaine was detected after decontaminating the samples. LC-MS/MS detected 5 ng/10 mg in the sample after the washing. Finally, the authors concluded that the washing protocols are not reliable, because external cocaine can migrate into the hair, and recommended a simple analysis of images which makes the evaluation of the differences among hair samples contaminated externally and the interpretation of the correct results easier [43].

#### 3.1.4. Other Methods of Ionization

Paper spray (PS) technique was introduced in 2009 and has been used in the development of a wide range of quantitative and qualitative applications. Here, the sample is deposited in the paper with a sharp point, and ions are produced by voltage applied, while the substrate is held by a metal clip in the paper and placed in the front of the inlet of a mass spectrometer. Then, the front mass spectrometer performs the detection after the sample elution, which can be carried out in the same manner of paper chromatography, but with a direct sample injection to the mass spectrometer (Figure 2(f)). In this technique, a wide range of chemicals can be ionized by paper spray, from small molecules to large biomolecules [8].

Paper spray ionization coupled to high-resolution tandem mass spectrometry (PSI-HR-MS/MS) have also been used in order to validate a screening of drugs in urine such as codeine-6-glucuronide, diclofenac, among others, and to validate a comprehensive urine screening. Nevertheless, the procedure showed high matrix effects for most drugs, but also acceptable limits of identification that have the potential of reducing workload. However, the authors recommend its implementation as a promising alternative to conventional procedures, but they warned that there is a risk of false positive/negative results caused by mixed spectra during the detection of low concentrations. Therefore, some problems should be solved before implementing it in routine analysis [44].

Thin-layer chromatography (TLC) has been also used as an introductory sampling method combined with PS-MS to analyze cocaine and its adulterants in 10 *µ*L of sample. This analysis obtained promising results in which the limit of detection was reduced five thousand times (1.0 *μ*g/mL), showing an *R*^2^ > 0.999 that is another indicator of the reliability of this technique, and the possibility to be implemented in routine analyses [45]. In the same manner, simultaneous analyses of methamphetamines, cocaine, morphine, and Δ^9^-tetrahydrocannabinol were performed in a single blood spot by PS-MS in only 2 minutes, with minimal sample preparation through the extraction of the compounds by solvents [46].

PS-MS has also been used in positive ionization mode to obtain chemical profiles of illicit drugs such as blotter papers containing extracts and leaves of natural cannabinoids and synthetic cannabinoids; here, 1 mg of blotter paper was used as the PS ionization source. For this reason, the authors recommend to be careful with the low sensitivity of this technique that was observed to possibly occur due to an ionic suppression process, caused by the matrix effect (extracted impurities from the surface of the blotter paper). The results provided a limit of detection of around 0.17 ppb [47].

PS-MS/MS has been used in targeted drug screening using an Orbitrap QMS, one in positive mode and the other in negative mode. In the positive ion mode, over 130 drugs and drug metabolites in postmortem samples were semiquantitatively determined, proving an adequate method in postmortem analysis. In the analysis in negative mode, an ion-screening method was also developed for a small panel of barbiturates and structural analogs. This method showed good qualitative agreement with LC–MS-MS; the true positive rate of paper spray MS/MS was 92%, and the true negative rate was over 98%. This result shows that this technique possesses the necessary potential for acidic drug detection and screening without sample preparation; however, the authors did not present a list of possible interferences during the analysis [48].

Most recently, PS-MS has been used modifying the paper through molecularly imprinted polymers (MIP) to create a specific site for cocaine analysis in 1 mL of the oral fluid. In this case, the PS was set by holding the membrane connected directly to the outlet probe of the ESI with a 0.5 mm wire using an alligator-type clip and applying a voltage of 4 V, obtaining an LOQ of 100 ng/mL, and becoming a promising method to analyze cocaine [49].

High-performance ion mobility spectrometry (HPIMS) has been used along with electrospray ionization to detect codeine and morphine in urine samples without extra sample pretreatment (Figure 2(g)). However, issues of charge suppression in the presence of drug mixtures interfering with matrix components were observed, so the authors recommended considering some previous steps before sample preparation. For instance, the authors introduced a sample into a drift tube via pulse Bradbury–Neilson ion gate and operated it in positive mode, and the ions passed to desolvation to be separated [50]. This method achieved a resolving power double than the currently accepted method without an excessive necessity of sample preparation [51].

Ion mobility-based separation methods can be combined with mass spectrometry (IMS-MS) in order to minimize chemical suppression caused by interference and the use of chromatography separations to targeted applications (Figure 2(h)). The interface has only a few centimeters in length and operates in seconds; besides, it can be adapted to any MS system using atmospheric-pressure ionization-targeted applications. In this analysis, a miniature differential ion mobility filter is used and placed in front of the entrance of the mass spectrometer, and a solution of 10 ng/mL of the sample was introduced using infusion introduction of ions created by electrospray ionization source coupled with ion trap MS/MS. This method allowed the characterization of samples in 30 seconds, reducing case backlogs in the targeted analysis of analytes of interest, showing the range of quantification of around 0.01–10 ng⁄uL of cocaine [52].

Recently, a new method has been developed coupling microfluidics with a miniature mass spectrometer in order to quantify cocaine in urine samples. This method is able to deliver droplets of solvents to dried urine samples, separating droplets of 80 *µ*L of extracts, then performing splits from the hydrophilic dried urine zones and driving them to the destination electrode for analysis. The LOQ for cocaine was 40 ng/mL [74].

Another recent method of direct analysis is touch spray (TS). In this technique, the sample is transferred to a substrate with subsequent ionization; in this manner, the substrate can serve both as the means for the sample collection, ionization, and as straightforward handling analysis of either solid or liquid samples without pretreatment (Figure 2(i)). Using TS-MS coupled with MS/MS, drugs of abuse like Δ^9^-tetrahydrocannabinol and buprenorphine were identified in spiked oral fluid using medical swabs directly, providing limits of detection of around 50 ng/mL, which are sought by international forensic and toxicological societies. This adaptation of medical swabs for TS-MS analysis allows noninvasive and direct sampling of neat oral fluids; however, the authors affirm that the drying step represents the most time-consuming part of the analytical protocol, but the potential of the technique is high in terms of specificity, selectivity, and sensitivity [9].

More recently, laser diode thermal desorption (LDTD) and atmospheric solids analysis probe (ASAP) have been coupled with HRMS using APCI ionization in order to generate high-quality data from multiple samples with none or minimal sample preparation, with the purpose of identifying synthetic cannabinoids/cathinones through full-MS and MS/MS experiments. In ASAP, a melting-point capillary tube is used to introduce the sample into a stream of heated nitrogen gas, which results in the sample being desorbed from the capillary [53], and the desorbed sample is then ionized by a corona discharge needle. During ASAP-MS analysis, it was possible to examine solid and liquid samples transferred to the capillary surface (Figure 2(j)); whereas in the LDTD-MS analysis, the samples were extracted by a solvent. This method uses a specially designed 96-well plate with stainless alloy steel inserts, where the sample is thermally desorbed from the stainless steel by an infrared laser which forms neutral gas-phase molecules [54] (Figure 2(k)). These gas-phase molecules are carried into the mass spectrometer inlet by compressed air. Before they enter the mass spectrometer inlet, a corona discharge needle ionizes the neutral molecules.

This LDTD-APCI-MS method results in a completely automated analysis with low sampling times. The authors recommended the use of both methods of ambient ionization, which allow rapid experiments from a single sample introduction. However, when performing the optimization, they verified that the simplicity of ASAP design allows it to be easily switched between API techniques and possible positive/negative switching for a single sample introduction, which provides many possibilities of optimization during the analysis. More studies in this field are required, especially in possible interference of suppression of ionization [75].

## 4. Conclusions

Mass spectrometry is the most important technique used in toxicological forensic analysis. MS coupled with chromatography are the preferred techniques to identify new drugs or metabolites through screening analysis, providing excellent results in limit of detection, precision, accuracy, and sensitivity, although it may be a time-consuming process. Direct techniques with MS (with less sample preparation) are more likely to be used in target analysis or in routine qualitative analysis. However, sample complexity complicates the identification among compounds with similar fragmentation patterns, along with the problems caused by ionization chemical suppression. As a result, recent developments in MS are concerned with the necessity of creating new software in order to help improve simplicity and robustness in the identification of drugs. There is a growing necessity to develop more innovative methodologies to reduce time consumption in the analyses, enhance sensitivity, and finally move forward towards greener chemistry.

## Figures and Tables

**Table 1:** Main modifications and modes of analysis applied in mass spectrometry in forensic toxicological analysis.

Ionization techniques in mass spectrometry coupled to separations techniques			
Type of MS analysis	Advantages	Disadvantages	References
Negative chemical ionization	(i) It provides more sensitivity at low concentration (pg) based on the stability of electronegative moieties.	(i) Better results are provided when the technique is combined with EI-MS in order to obtain more structural information.	[14]
	(ii) Avoids wrong interpretations of correct results reducing time consumption.	(ii) This method requires an additional reagent for the ionization; methane is commonly used.	

Cold electrospray ionization	(i) It can be considered as a miniaturized analytical method because of the interface that it uses and the supersonic molecular beams through analysis with short columns and high column flow rates.	(i) Additional instrumentation is required.	[15, 16]
	(ii) Can provide enhanced molecular ions to much larger and more polar compounds with GC, using the same library to EI-MS (NIST).		
	(iii) The flow rate can be increased up to 100 mL/min, and its fly-through ion source sensitivity is fully independent from the column flow rate.		
	(iv) In this method, the nozzle flow rate is constant; as a result, the cold EI fly-through ion source is unaffected by the column flow rate, unlike any other ion source.		
	(v) The use of GC-MS with cold EI has no limitations for the column used.		

Surface-activated chemical ionization	(i) The ionization of solutes occurs upon the polarization of neutral, solvent molecules, which makes it a highly sensitive method.	(i) SACI is used to maximize the sensitivity in the analysis of highly polar compounds, but data about less polar compounds have not been revealed until now.	[17]
	(ii) The electrostatically charged surface increases the ESI ionization efficiency.		
	(iii) When it is used with ESI, the efficiency of proton-transfer ionization reactions is enhanced by the polarization of neutral solvent molecules or by charged solute molecules induced by the proximity of the charged surface.		
	(iv) The solvent and the analyte ions are better focused towards the analyzer.		
	(v) The increase in signal intensity provides an increase in sensitivity, because there is a reduction in the chemical noise observed in the mass analyzer.		
*New methods of analysis used in mass spectrometry*			
Dynamic multiple reaction monitoring mode of analysis	(i) This method monitors the analytes only around the expected retention time, decreasing the number of concurrent MRM transitions, allowing both the cycle and the dwell time, which can be optimized in order to obtain higher sensitivity, accuracy, and reproducibility.	(i) It is necessary to maintain the analyte analysis in the same polar mode since a switch of polarity within a single run would reduce the sensitivity and accuracy of quantification with the applied MS instrumentation.	[17–20]
	(ii) dMRM allows the monitoring of more MRM transitions in a single run without compromising data quality.	(ii) The retention time must be informed, optimized, and defined with reference standards using established chromatographic conditions if it is possible. If the retention time drifts, this might result in an incomplete peak definition and quantitation.	
	(iii) The dwell time is intelligently optimized by association with the delta retention time. Additionally, information about delta retention time and retention time are key to maximize the dwell time and increasing sensitivity.	(iii) It is necessary to optimize the MS conditions for the all transitions.	
	(iv) This method gives the possibility of applying simultaneous quantifications of multiple components.		

*Ambient ionization techniques in mass spectrometry*			
Desorption electrospray ionization-mass spectrometry	(i) Direct analysis with high-velocity nebulizing gas.	(i) During analysis of drugs in biological matrix with a high amount of salt, the suppression ionization effect is elevated.	[8, 21–23]
	(ii) The selectivity and sensitivity of this technique can be increased by a pretreatment sample.	(ii) The ion source geometry affects the dynamic of the splashing mechanism resulting in changes in droplet size, charge, and analyte dissolution extent.	
		(iii) A high velocity of nebulization can mechanically ablate delicate samples/powders.	

Desorption atmospheric-pressure photoionization	(i) Matrix with high salt content do not provide an elevated suppression of ionization.	(i) High suppression ionization can be found depending of the biological matrix.	[8, 21–25]
		(ii) Sample preparation is commonly needed in order to avoid suppression of ionization.	

Direct analysis in real time	(i) It is commonly used in the analysis of drugs of low molecular weight; therefore, its sensitivity depends on analyte volatility.	(i) Compounds of high molecular weight may need derivatization.	[4, 8, 11, 26–35]
	(ii) The geometrical configuration of the ion source is simple and robust for its operation.	(ii) Its sensitivity depends of the temperature of the ionization region; therefore, the higher the temperature is the higher the risk of damage is.	
	(iii) Pretreatment of sample can increase the selectivity of the analysis in complex biological samples.	(iii) Its reproducibility depends on the position of the sample inside the ion source, which represents a big problem in the quantification of the analysis.	
Low-temperature plasma	(i) It is possible to perform direct analysis without sample preparation.	(i) This technique is exclusively used with small organic molecules with low to moderate polarity.	[8, 36]
	(ii) The instrumentation is simple, and its configuration provides low consumption of discharge gas and the possibility of using air as the discharge gas.		
	(iii) High sensitivity and sensitivity can be obtained without pretreatment of the samples.		

Matrix-assisted laser desorption electrospray ionization	(i) It can be coupled to mass spectrometric imaging (MSI) in order to obtain the distribution spectra of the target.	(i) Quantitative analysis has not been carried out until this present date.	[8, 37–41]
	(ii) A mode of analysis called “dynamic pixel” can be used to obtain an imaging method that is faster to do a screening of the compounds.		
	(iii) The analysis does not need sample preparation. This method is based on a direct analysis over the sample.		
	(iv) The sensitivity of the analysis can be improved using a specific matrix. For example, umbelliferone matrix obtained better results in the analysis of methamphetamine in hair than the common matrices CHCA or DHB.		
	(v) The technique has been tested along with MAMS, and it is possible to cause reproducibility of the signal with this technology.		

Metal-assisted secondary ion mass spectrometry	(i) It can be coupled to mass spectrometric imaging (MSI) in order to obtain the distribution spectra of the target.	(i) Quantitative analysis has not been carried out.	[8, 42, 43]
	(ii) The limits of detection are lower than those obtained with MALDESI and also compared with the ones with LC-MS/MS.		
	(iii) It is not necessary to perform preparation of the sample.		

Paper spray	(i) This technique can analyze a wide range of molecules, from small to large biomolecules.	(i) It has a high matrix effect on most of the drugs.	[8, 44–51]
	(ii) The use of a pretreatment of the sample can enhance the sensitivity of the analysis.	(ii) The paper can extract impurities from the surface and cause the suppression of ionization.	

High-performance ion mobility spectrometry	(i) Methods of introduction of samples such as a chromatographic separation can be used to minimize the suppression of ionization.	(i) Direct analysis can result in suppression of ionization.	[50, 51]
Differential mobility spectrometry-mass spectrometry separation	(i) Separation conditions of the target analysis can be selectively transmitted into a mass spectrometer.	(i) This technique is rarely being implemented in commercial devices, and it is not known yet whether it can be used to establish profiles of drug mixtures in complex biological samples.	[52]
	(ii) It can be considered as an ionization technique coupled to a separation method that has a small interface which gives results in few seconds.		

Touch spray	(i) The substrate (medical swabs) used can serve as a sample collection tool; thus, ionization helps in the analysis of solid or liquid samples without pretreatment.	(i) The drying step of this substrate represents the most time-consuming part of the analytical protocol.	[9]
	(ii) The TS-MS can allow noninvasive and direct sampling of neat oral fluids.		

Laser diode thermal desorption	(i) The method is completely automatic.	(i) It is not possible to perform a simple interchange between negative and positive modes of ionization.	[53]
		(ii) Effects of interferences in complex biological samples must be explored, and more sample preparation is necessary before the liquid samples are transferred towards the capillary surface.	

Atmospheric solids probe analysis	(i) It is possible to perform analysis of solids and liquids easily.	(i) Effects of interferences in complex biological samples must be explored with more detail.	[53, 54]
	(ii) This design allows the possibility of positive/negative switch during the analysis.	(ii) This technique provides better sensitivity during the analysis of small-molecule drugs, decreasing the analysis of the high-molecule compounds.	
